# Experimental Investigation of CO_2_ Uptake and Absorption Kinetics in APTES-Modified Fe_3_O_4_/Water Nanofluids

**DOI:** 10.3390/nano16211404

**Published:** 2026-11-01

**Authors:** Seokhyun Ko, Muhammad Ali, Jin Hyun Kim, Honghyun Cho, Jeonggyun Ham

**Affiliations:** 1 Department of Mechanical Engineering, Chosun University, 10, Chosundae 1-gil, Dong-gu, Jeonnam-Gwangju 61452, Republic of Korea; rhrh782@naver.com (S.K.); hhcho@chosun.ac.kr (H.C.); 2 Department of Mechanical Engineering, Graduate School of Chosun University, 10, Chosundae 1-gil, Dong-gu, Jeonnam-Gwangju Special Metropolitan City 61452, Republic of Korea; ali99479@chosun.ac.kr; 3 Cooperation Foundation, Sunchon National University, Suncheon-si, Jeonnam-Gwangju 57922, Republic of Korea; apc615k@scnu.ac.kr

**Keywords:** absorption kinetics, CO_2_ absorption, effective CO_2_ uptake, APTES-modified Fe_3_O_4_ nanofluid, gas–liquid mass transfer

## Abstract

This study experimentally investigated CO_2_ absorption in APTES-modified Fe_3_O_4_/water nanofluids under continuous bubbling at nanoparticle concentrations of 0–0.1 wt% and temperatures of 10, 20, and 30 °C. Performance was evaluated using effective CO_2_ uptake and the apparent absorption rate constant, with enhancements calculated relative to deionized (DI) water at the same temperature. Effective uptake decreased with increasing temperature and exhibited a nonmonotonic concentration dependence, whereas normalized uptake profiles generally indicated a more rapid approach to the final uptake at higher temperatures. The highest uptake was 2.084 g L^−1^ at 10 °C and 0.01 wt%. The largest uptake enhancement was approximately 13.2% at 30 °C and 0.05 wt%, although the apparent rate constant decreased by approximately 4.1% under this condition. The greatest kinetic enhancement occurred at 10 °C and 0.1 wt%. Enhancement ranges across concentrations widened with increasing temperature for both metrics, while the largest ranges across temperatures occurred at different concentrations for uptake and kinetics. Normalized performance maps showed that conditions favoring uptake did not necessarily maximize the kinetic benefit of nanoparticle addition. These results support selecting nanoparticle concentration in relation to operating temperature and the relative importance of uptake and absorption rate.

## 1. Introduction

The continuous increase in atmospheric CO_2_ concentration represents a major environmental and energy challenge for climate-change mitigation, and further advancement of carbon capture, utilization, and storage (CCUS) technologies is required to reduce CO_2_ emissions from large stationary sources such as power plants, cement plants, and steelworks [1,2]. Liquid-phase absorption is widely used in industrial processes because of its high operational flexibility and capture efficiency; however, conventional amine-based absorption processes involve substantial thermal energy requirements for solvent regeneration as well as operational burdens associated with solvent degradation and equipment corrosion [2,3]. Accordingly, nanofluid-based CO_2_ absorption has been actively investigated as a process-intensification strategy that can enhance gas–liquid mass transfer and improve absorbent utilization while minimizing structural modifications to existing absorption equipment [1,2,3].

Early studies on nanofluid-assisted CO_2_ absorption mainly focused on dispersing metal-oxide nanoparticles in physical absorbents such as water or methanol to enhance gas–liquid mass transfer. Kim et al. [4] reported an increase in the mass-transfer coefficient during CO_2_ absorption in methanol/Al_2_O_3_ nanofluids, while Torres Pineda et al. [5] and Kim et al. [6] analyzed bubble-absorption behavior and suggested that suspended nanoparticles can affect liquid-side mass transfer and interfacial renewal. Subsequent studies have discussed Brownian-motion-induced micro-convection, the grazing or shuttle effect by which particles transport dissolved species from the interface into the bulk liquid, surface renewal caused by boundary-layer disturbance, and changes in bubble formation, detachment, and coalescence as possible mechanisms of mass-transfer enhancement [3,5,6,7]. More recently, Cheng et al. [8] directly visualized bubble behavior in a bubble column using high-speed imaging and showed that nanoparticles can shorten bubble detachment time, reduce detachment diameter, and increase bubble generation frequency, thereby modifying the effective gas–liquid interfacial area. These findings indicate that nanofluid effects can arise not only from changes in solubility but also from modifications of interfacial and hydrodynamic behavior.

Research has subsequently expanded from physical absorption systems to reactive absorption systems. Pashaei et al. [9] and Pashaei and Ghaemi [10] investigated metal-oxide nanoparticles dispersed in aqueous piperazine and diethanolamine solutions and demonstrated the potential for mass-transfer enhancement in reactive absorbents. Kim et al. [11,12] employed dodecane nanoemulsions to exploit a shuttle effect in which the dispersed phase temporarily absorbs and transports CO_2_, whereas Boldoo et al. [13] combined an Al_2_O_3_/methanol nanoabsorbent with porous Ni foam to increase both gas–liquid contact and the effective transfer area. Thus, nanofluid research has progressed from optimization of nanoparticle type toward control of interfacial behavior, integration with reactive absorbents, and combination with porous structures for process intensification.

Fe_3_O_4_ has been continuously investigated as a nanoparticle for CO_2_ absorption because it offers both favorable dispersibility and recoverability in aqueous media and versatile surface functionalization. Ansarian and Beiki [14] reported a maximum absorption enhancement of approximately 13.4% using 0.005–0.05 vol% Fe_3_O_4_/water nanofluids in a bubble column and observed a nonlinear dependence on nanoparticle concentration. Peyravi et al. [15] compared Fe_3_O_4_, CNT, SiO_2_, and Al_2_O_3_ nanofluids in a hollow-fiber membrane contactor and reported an approximately 43.8% increase in the liquid-side mass-transfer coefficient at 0.15 wt% Fe_3_O_4_, emphasizing dispersion stability and hydrodynamic diameter as important parameters governing absorption performance. Pahlevaninezhad et al. [16] reported maximum mass-transfer-coefficient enhancements of 111% and 13.7% under turbulent and laminar conditions, respectively, for a 0.05 vol% Fe_3_O_4_/water falling-film system. Karimi Darvanjooghi et al. [17] further reported increases in CO_2_ molar flux, mass diffusivity, and surface-renewal factor together with a reduction in diffusion-layer thickness, indicating that Fe_3_O_4_ nanoparticles can modify dynamic gas–liquid transfer characteristics.

Recent studies have increasingly focused on modifying the surface chemistry of Fe_3_O_4_ to improve CO_2_ affinity and aqueous dispersibility in addition to its intrinsic mass-transfer-promoting effect. Arshadi et al. [7] reported up to approximately 70.3% enhancement in CO_2_ absorption using aqueous nanofluids containing SiO_2_- and amine-functionalized Fe_3_O_4_, while Elhambakhsh and Keshavarz [18] reported up to approximately 34.2% enhancement in absorption capacity in distilled water using functionalized magnetic nanoparticles including Fe_3_O_4_@SiO_2_–NH_2_. Hafizi et al. [19] and Elhambakhsh et al. [20] also reported improved absorption/desorption performance using magnetic nanoparticles functionalized with amine- or amino-acid-based groups. More recently, Islam et al. [21] investigated amine-functionalized silica-coated magnetic iron oxide for aqueous CO_2_ adsorption and comprehensively characterized crystallinity, surface functional groups, morphology, and dispersion behavior using XRD, FTIR, TEM, and zeta-potential measurements. Collectively, these studies show that the performance of Fe_3_O_4_-based nanofluids is governed not only by nanoparticle concentration but also by surface modification and dispersion state.

Collectively, previous studies demonstrate that Fe_3_O_4_-based nanofluids can promote CO_2_ transfer in diverse absorption systems, although the reported degree of enhancement varies substantially with apparatus configuration, operating pressure, external-field application, nanoparticle concentration, and surface-modification strategy [1,3,22]. Magnetic-field-assisted falling-film and single-bubble systems [16,17], high-pressure batch absorbers [18], hollow-fiber membrane contactors [15], and aqueous or amine-based absorbents containing functionalized Fe_3_O_4_ particles [7,19] have been examined under markedly different hydrodynamic and mass-transfer environments, which limits direct comparison on a common basis. Thus, despite previous investigations of functionalized Fe_3_O_4_ nanoparticles [7,18,21], systematic comparisons of effective CO_2_ uptake and apparent absorption kinetics across nanoparticle concentrations and temperatures under identical continuous bubbling conditions remain limited.

This research gap is particularly relevant because temperature can affect CO_2_ uptake capacity and absorption kinetics in different directions. In physical CO_2_ absorption, increasing temperature generally decreases the CO_2_ carrying capacity of aqueous absorbents while potentially accelerating molecular diffusion and interfacial mass transfer [16,17,20,23,24]. Consequently, evaluation based solely on the final absorbed amount may not adequately reflect nanoparticle-induced changes in dynamic transport, whereas comparison based only on absorption rate does not describe the amount of CO_2_ retained during operation. Effective CO_2_ uptake and absorption kinetics therefore need to be evaluated separately under identical concentration–temperature conditions, with nanoparticle-induced changes quantified relative to DI water at the same temperature.

In this study, Fe_3_O_4_ nanoparticles synthesized by co-precipitation were subsequently treated with APTES to prepare APTES-modified Fe_3_O_4_ nanoparticles, which were then dispersed in water to prepare low-concentration nanofluids. Nanoparticle concentration was varied from 0 to 0.1 wt%, and CO_2_ was continuously supplied at a constant flow rate at 10 °C, 20 °C, and 30 °C to compare concentration- and temperature-dependent absorption behavior. Effective CO_2_ uptake and the apparent absorption rate constant were used as respective indicators of uptake capacity and absorption kinetics, allowing the concentration–temperature dependence of the two performance metrics to be quantified separately. The objective was to establish a systematic description of CO_2_ absorption in low-concentration APTES-modified Fe_3_O_4_/water nanofluids and to provide quantitative information relevant to selecting operating conditions for nanofluid-based absorption processes.

## 2. Materials and Methods

### 2.1. APTES-Modified Fe_3_O_4_/Water Nanofluids

Fe_3_O_4_ nanoparticles were synthesized using an HCl-protected co-precipitation method without an inert atmosphere. Extra-pure-grade FeCl_2_·4H_2_O (SAMCHUN, Republic of Korea), FeCl_3_·6H_2_O (SAMCHUN, Republic of Korea), HCl (DAEJUNG, Republic of Korea), and aqueous ammonia were used as the starting reagents. FeCl_2_·4H_2_O and FeCl_3_·6H_2_O were separately dissolved in 2 M HCl to prepare 1.0 M FeCl_2_ and 2.0 M FeCl_3_ precursor solutions, respectively. The prepared solutions were mixed at an Fe^3+^:Fe^2+^ molar ratio of 2:1 under continuous stirring at 150 rpm, following the stoichiometric ratio required for Fe_3_O_4_ formation. The precursor mixture was then introduced into 40 mL of distilled water preheated to 70–80 °C. Subsequently, 0.7 M aqueous ammonia (SAMCHUN, Republic of Korea), was rapidly added until the solution pH reached approximately 10, resulting in the formation of a black Fe_3_O_4_ precipitate. The reaction mixture was then aged at 50–60 °C for 20–30 min to promote nanoparticle formation and growth. After completion of the reaction, the Fe_3_O_4_ nanoparticles were collected using an external magnet and washed three times with ethanol (DAEJUNG, Republic of Korea) to remove residual ions and unreacted reagents. The overall precipitation reaction can be expressed as Equation (1).

(1) $$2Fe{Cl}_{3}+Fe{Cl}_{2}+8{NH}_{4}\to {{Fe}_{3}O}_{4}+8{NH}_{4}Cl$$

**Figure 1:**
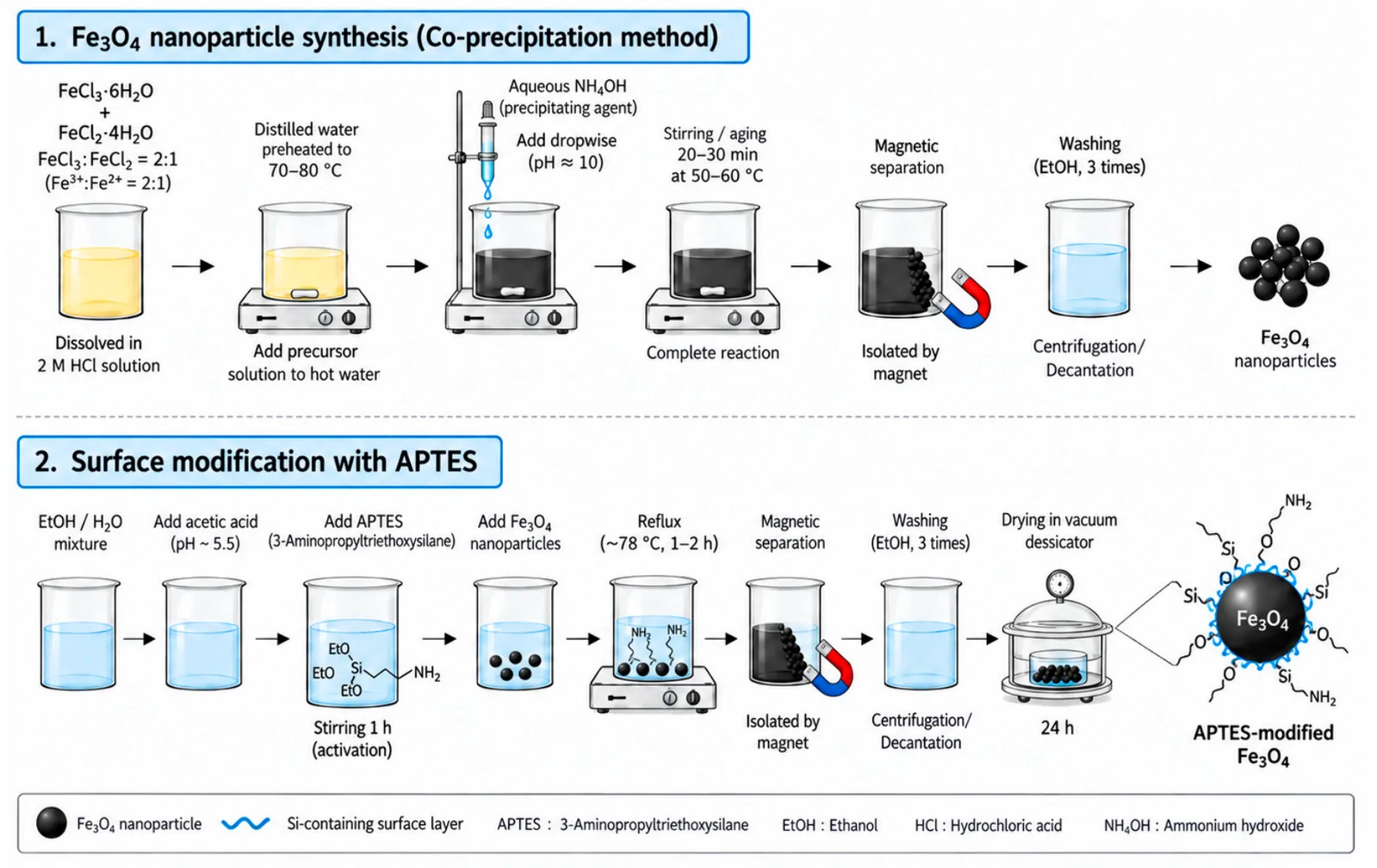
Schematic illustration of the synthesis and APTES modification of Fe_3_O_4_ nanoparticles.

Figure 1 illustrates the synthesis of Fe_3_O_4_ nanoparticles and the subsequent SiO_2_ surface-modification procedure. Step 1 shows the co-precipitation synthesis of Fe_3_O_4_, whereas Step 2 shows the APTES-assisted silanization process used for surface modification.

Surface modification of Fe_3_O_4_ nanoparticles was performed through treatment with APTES (Sigma-Aldrich, America). Acetic acid was added to an ethanol–distilled-water mixture to adjust the pH to 5.5, after which APTES was introduced and the solution was stirred for 1 h. Fe_3_O_4_ nanoparticles were then added to the activated solution and allowed to react at 50–60 °C for 1–2 h. After the reaction, the particles were magnetically recovered, washed three times, and dried in a vacuum desiccator for 24 h to obtain APTES-modified Fe_3_O_4_ nanoparticles.

**Figure 2:**
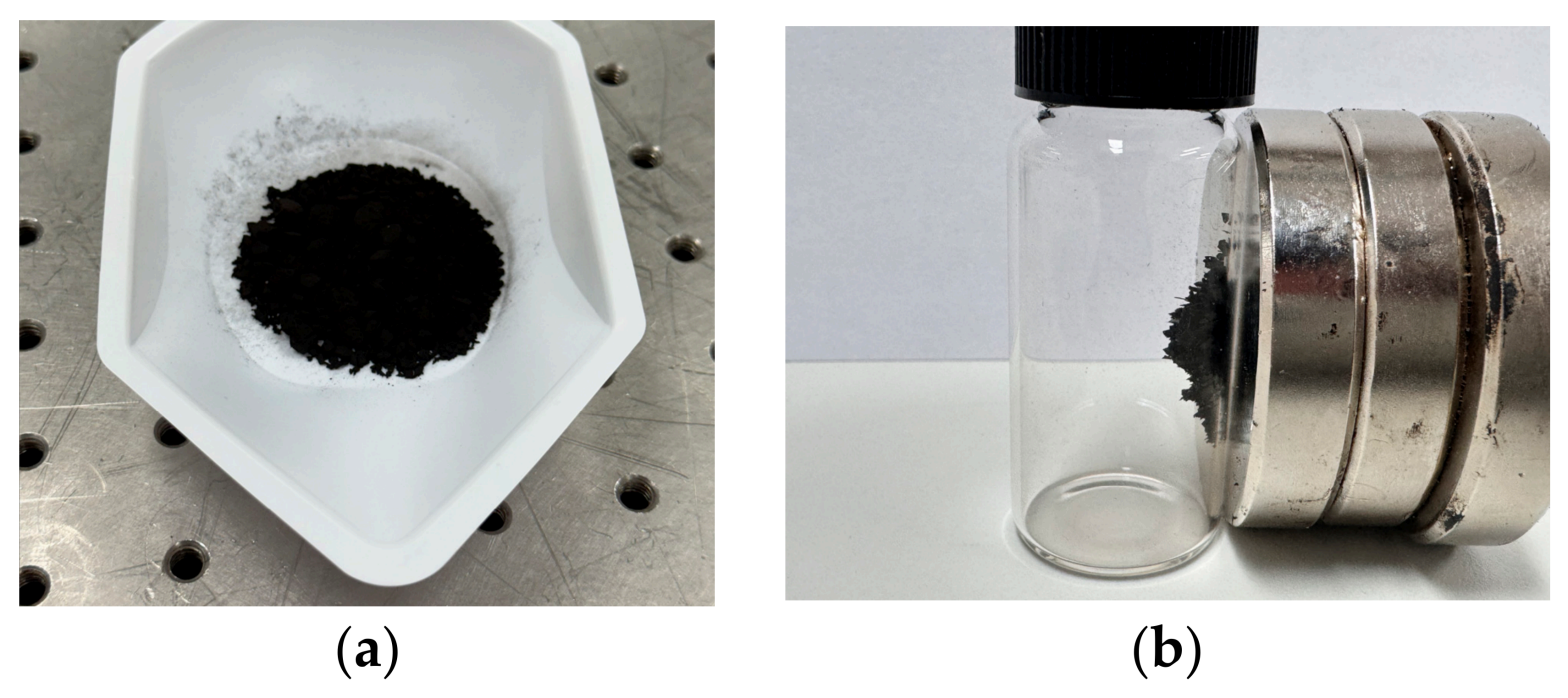
Photographs of (**a**) synthesized APTES-modified Fe_3_O_4_ nanoparticles and (**b**) magnetic attraction of APTES-modified Fe_3_O_4_ nanoparticles under an external magnetic field.

APTES-modified Fe_3_O_4_ nanoparticles were dispersed in 600 mL of ultrapure water to prepare nanofluids at concentrations of 0, 0.005, 0.01, 0.05, and 0.1 wt%. Dispersion was performed using a horn-type ultrasonic homogenizer (STH-750S, Sonictopia, Republic of Korea) for 2 h in a double-jacketed beaker, with the nanofluid temperature maintained at 20 °C using a thermostatic circulator. Figure 2 shows the synthesized nanoparticles and prepared nanofluids. The zeta potential of the 0.01 wt% nanofluid was −32 mV at pH 5.16, indicating favorable electrostatic dispersion stability under the measurement conditions. In addition, no visible sedimentation was observed during one week of storage.

### 2.2. Characterization of Nanoparticles and Nanofluids

The crystalline structure, morphology, and elemental composition of the APTES-modified Fe_3_O_4_ nanoparticles were investigated using X-ray diffraction (XRD; X’Pert PRO MPD, PANalytical, Almelo, The Netherlands) and scanning electron microscopy coupled with energy-dispersive X-ray spectroscopy (SEM/EDS; FE-SEM SU8600, Hitachi High-Tech Corporation, Japan). XRD measurements were performed using Cu Kα radiation (λ = 1.5406 Å) over a 2θ range of 3–90°. The average crystallite size was estimated from the full width at half maximum (FWHM) of the representative diffraction peak using the Scherrer equation:

(2) $$D=\frac{K\lambda }{\beta cos\theta }$$

**Figure 3:**
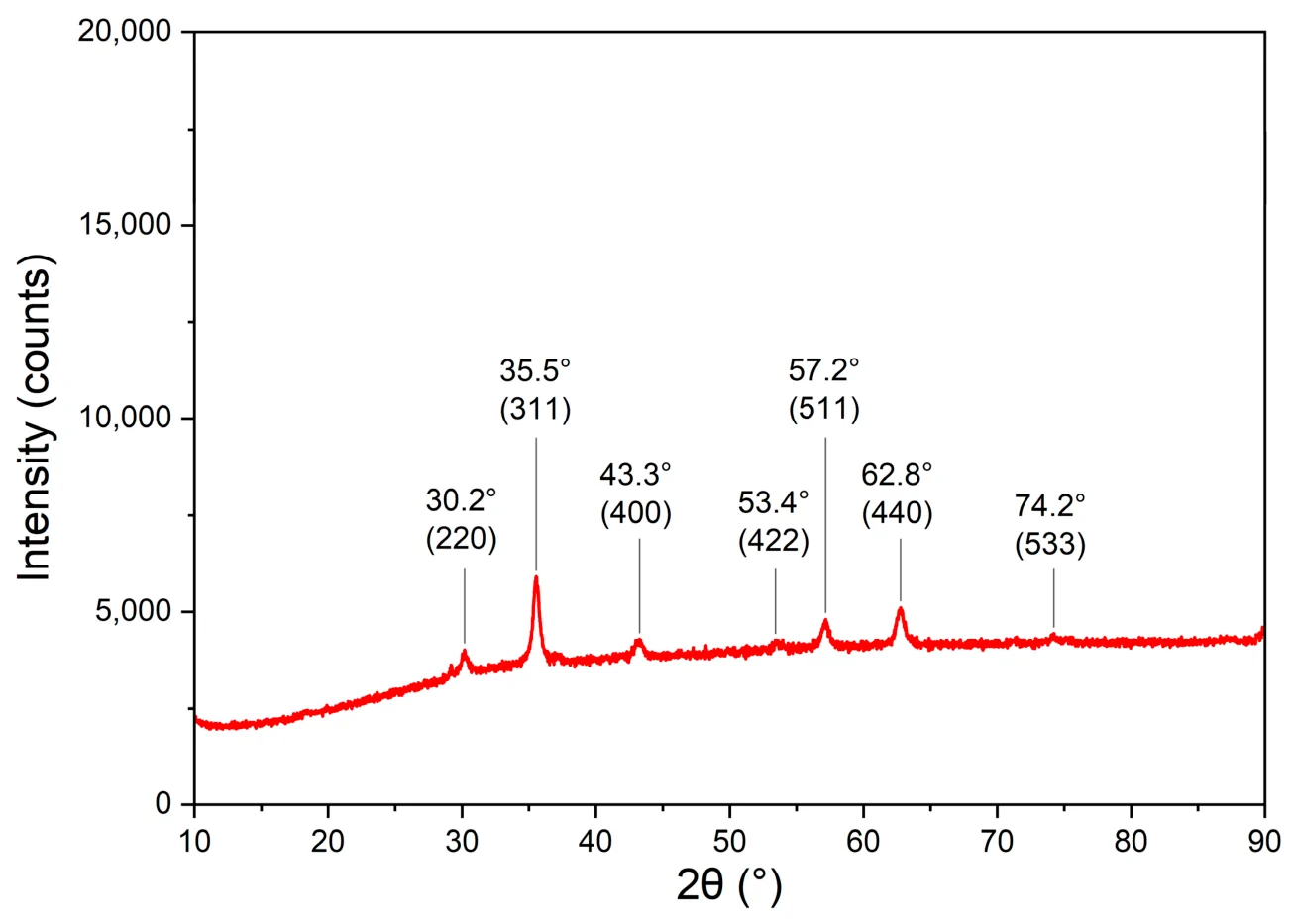
XRD pattern of APTES-modified Fe_3_O_4_ nanoparticles.

Figure 3 shows the XRD pattern of the synthesized APTES-modified Fe_3_O_4_ nanoparticles. The characteristic diffraction peaks were observed at 2θ = 30.14°, 35.51°, 43.08°, 53.47°, 57.17°, and 62.78°, corresponding to the (220), (311), (400), (422), (511), and (440) crystallographic planes, respectively. These diffraction peaks are consistent with the inverse-spinel structure of Fe_3_O_4_ (JCPDS No. 19-0629), supporting the formation of Fe_3_O_4_-based nanoparticles. The (311) reflection at 2θ = 35.51° exhibited the highest relative intensity. The crystallite size was estimated to be approximately 35.6 nm using the Scherrer equation with a shape factor of K = 0.9 and an FWHM of 0.2342° obtained from the (311) reflection of the sample.

**Figure 4:**
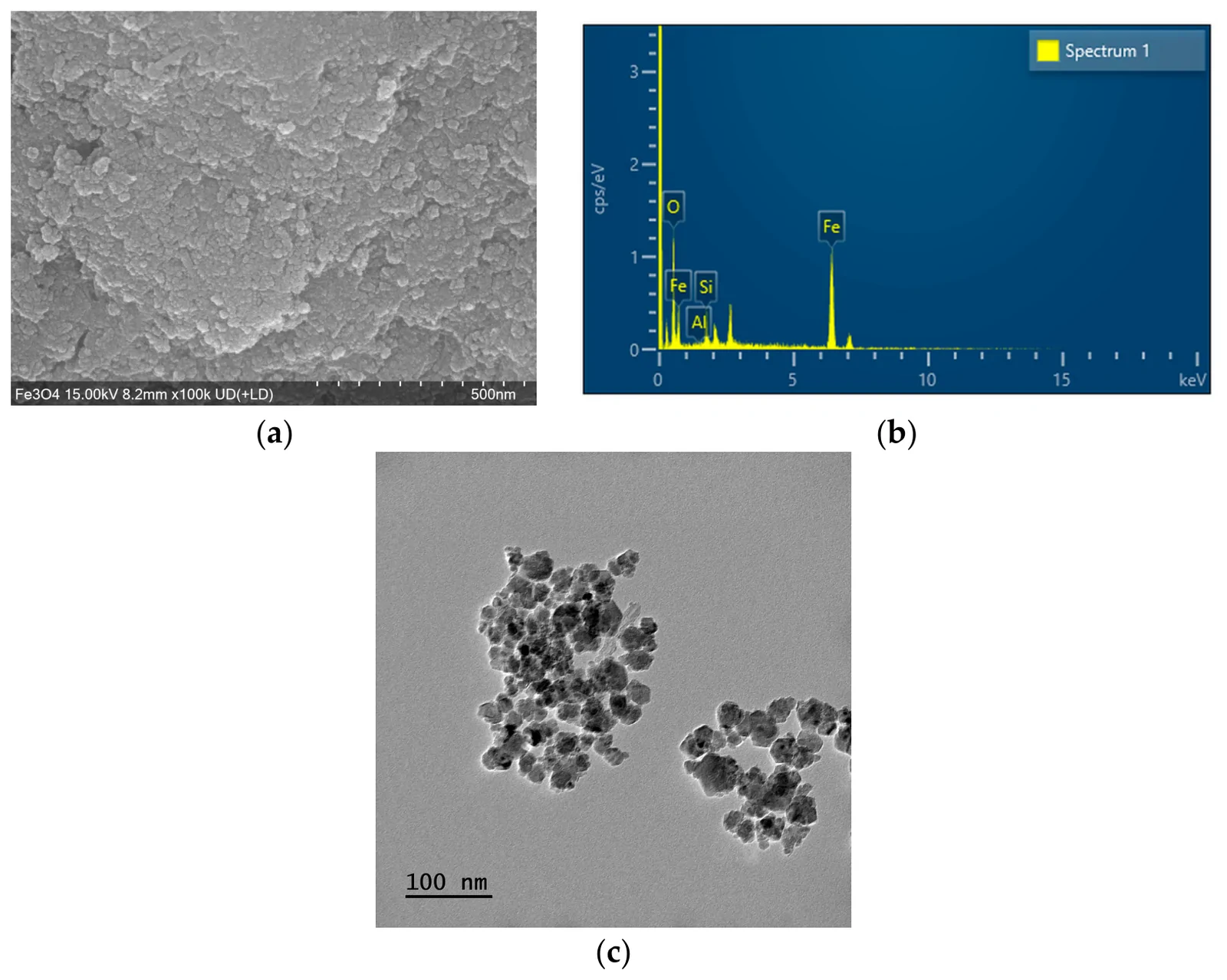
Morphological and elemental characterization of the synthesized APTES-modified Fe_3_O_4_ nanoparticles: (**a**) SEM image, (**b**) EDS spectrum and (**c**) TEM image.

Following the confirmation of the Fe_3_O_4_ crystalline phase by XRD, SEM and EDS analyses were conducted to examine the particle morphology and elemental composition of the APTES-modified Fe_3_O_4_ nanoparticles. Figure 4 presents the SEM image and the corresponding EDS spectrum. The SEM image reveals irregularly shaped particle aggregates with a rough surface morphology. The EDS spectrum exhibits distinct characteristic peaks of Fe, O, and Si. Semi-quantitative analysis indicates that the sample is composed of 68.30 wt% Fe, 27.49 wt% O, and 4.21 wt% Si, normalized to the total of these three elements. The presence of Si is consistent with the successful APTES-based surface modification of the Fe_3_O_4_ nanoparticles. A trace Al signal was also observed; this was attributed to the measurement setup and excluded from the quantification owing to its negligible contribution.

The morphology of the APTES-modified Fe_3_O_4_ nanoparticles was further examined using transmission electron microscopy (FE-TEM; JEM-2100F, JEOL Ltd., Akishima, Tokyo, Japan). The TEM image further reveals predominantly spherical to irregularly shaped nanoparticles with noticeable aggregation. The mean particle diameter and standard deviation were determined by image analysis of 156 identifiable nanoparticles in TEM images acquired during four imaging sessions (*n* = 156). The TEM-derived mean particle diameter was is in good agreement with the crystallite size of 35.6 nm calculated from the XRD data using the Scherrer equation, indicating that the nanoparticles are largely single-crystalline.

### 2.3. CO_2_ Absorption Apparatus and Procedure

**Figure 5:**
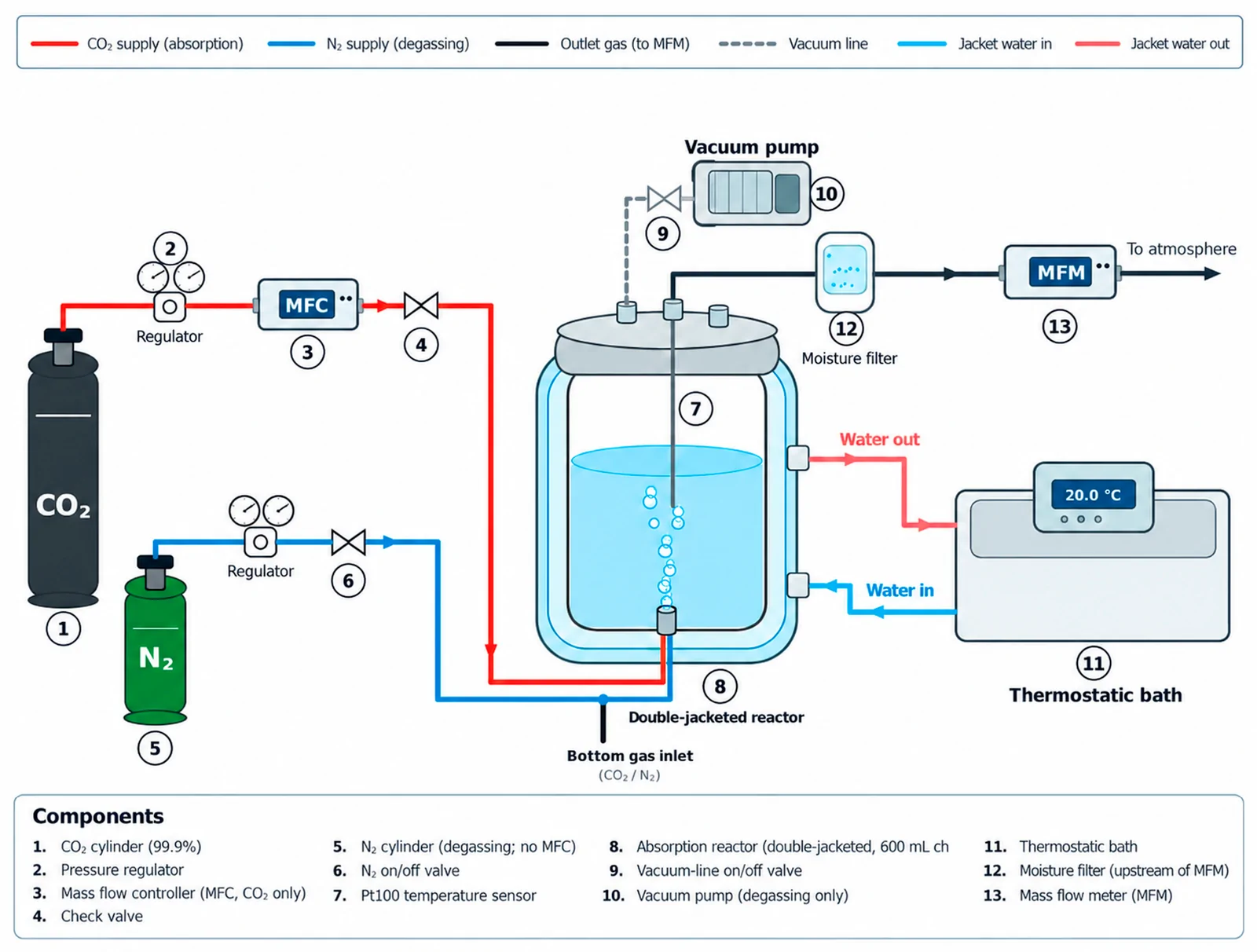
Schematic diagram of the CO_2_ absorption experimental setup.

The CO_2_ absorption experiments were conducted using a laboratory-scale gas–liquid absorption system designed to continuously monitor CO_2_ uptake from the difference between the inlet and outlet gas flow rates. A schematic diagram of the experimental apparatus is presented in Figure 5. The system consisted of a CO_2_ supply line, a flow-control section, a temperature-controlled absorption reactor, and an outlet gas-flow measurement line.

High-purity CO_2_ (99.9%) was supplied from a gas cylinder and delivered to the reactor through the gas-supply line. The gas flow rate was regulated using a mass flow controller (MFC), and a check valve was installed downstream of the MFC to prevent backflow of the absorbent into the gas-supply line. The controlled CO_2_ stream was introduced through a single-orifice bubble nozzle with a diameter of 4.57 mm located at the bottom of the reactor so that the gas dispersed into the liquid phase as bubbles and contacted the absorbent while rising through the column. No mechanical stirring was applied during the CO_2_ absorption experiments.

The absorption reactor was a cylindrical double-jacketed vessel with an inner diameter of 86 mm and a height of 158 mm. The absorbent was contained within the double-jacketed vessel, and temperature-controlled fluid was continuously circulated through the outer jacket using a thermostatic bath to maintain a constant absorbent temperature throughout each experiment. The liquid-phase temperature inside the reactor was monitored using a Pt 100 temperature sensor positioned in the absorbent. Prior to each experiment, the absorbent was degassed with N_2_ for 1 h to remove dissolved gases. During pretreatment, a vacuum pump was briefly used to reduce the reactor pressure to a mild vacuum level without inducing liquid evaporation. Following degassing, the reactor pressure was restored to atmospheric pressure under N_2_ purging. Consequently, the reactor headspace (approximately 0.3 L) contained predominantly N_2_ before CO_2_ injection, and all CO_2_ absorption experiments were conducted under atmospheric conditions. The gas leaving the upper part of the reactor passed through a moisture filter installed upstream of the mass flow meter (MFM). The outlet gas flow rate was then continuously measured by the MFM before discharge to the atmosphere.

For each experiment, the reactor was charged with 600 mL of DI water or APTES-modified Fe_3_O_4_/water nanofluid. Prior to the absorption experiments, preliminary flow verification tests were performed over the experimental flow range, confirming agreement between the flow rates measured by the MFC and MFM. The inlet CO_2_ flow rate was maintained at a nominal value of 40 sccm. The inlet CO_2_ flow rate was obtained from the measured flow-rate output signal of the MFC, rather than its set-point value, while the outlet flow rate was measured by the MFM. Both signals were continuously recorded and used to calculate CO_2_ uptake and absorption kinetics. The absorbent temperature was controlled at 10 °C, 20 °C, or 30 °C. The nanoparticle concentration and absorbent temperature were varied systematically to evaluate their effects on effective CO_2_ uptake and absorption kinetics. A fresh nanofluid sample was used for each experimental run.

### 2.4. Data Processing and Performance Metrics

The CO_2_ absorption performance was evaluated from the continuously measured inlet and outlet gas flow rates. The measured flow-rate data were used to quantify the instantaneous absorption behavior, cumulative CO_2_ uptake, and absorption kinetics under each operating condition. The inlet and outlet CO_2_ flow-rate data were processed using the actual sampling-time intervals. The instantaneous CO_2_ absorption efficiency was defined as

(3) $$\eta (t)=1-\frac{Q_{out}(t)}{Q_{in}(t)}$$

where $Q_{in}$ and $Q_{out}$ are the inlet and outlet standard volumetric flow rates obtained from the measured-flow output of the MFC and the MFM, respectively. A higher η indicates that a larger fraction of the supplied CO_2_ is transferred to the liquid phase. Each experiment was terminated when $Q_{out}/Q_{in}\geq$ 0.98.

The cumulative absorbed CO_2_ mass was calculated by trapezoidal integration of the inlet–outlet flow-rate difference:

(4) $$m_{abs}(t)\;=\;\frac{M_{{CO}_{2}}}{\;60V_{m,std}}\;\int_{0}^{t}\left(Q_{in}-Q_{out}\right)\;dt$$

where $M_{CO_{2}}$ is the molar mass of CO_2_ (44 g mol^−1^) and $V_{m,std}$ is the standard molar volume of an ideal gas (22,414 mL mol^−1^ at 0 °C and 1 atm). The factor 1/60 converts the time increment from seconds to minutes because the flow rates are expressed in sccm. As the MFC and MFM data were treated on a standard volumetric-flow basis, no additional temperature correction was applied to the measured flow rates. The effective CO_2_ uptake was determined from the cumulative absorbed mass at the termination point:

(5) $$q_{eff}\;=\;\frac{m_{abs,f}}{V_{L}}$$

where $V_{L}$ = 0.6 L. Here, $q_{eff}$ represents the operational CO_2_ uptake obtained under the common termination criterion rather than the thermodynamic equilibrium solubility.

To compare the dynamic absorption behavior independently of differences in final uptake, the cumulative absorbed mass was normalized as

(6) $$\theta (t)=\frac{m_{abs}(t)}{m_{abs,f}}$$

where θ ranges from 0 to 1. The apparent absorption rate constant, $k_{app}$, was used as an indicator of overall absorption kinetics rather than as an independently determined volumetric mass-transfer coefficient, $k_{La}$. The normalized uptake range of 0.2 ≤ θ ≤ 0.8 was selected to minimize the influence of initial transients and near-saturation behavior. Within this range, $k_{app}$ was obtained by linear regression using Equation (7):

(7) $$ln\left[1\;-\;\theta \left(t\right)\right]=a\;-k_{app}\;t\;(0.2\;\leq \;\theta \;\leq \;0.8)$$

where a is the regression intercept and $k_{app}$ is the negative of the regression slope. The coefficient of determination $R^{2}$ was used to assess the quality of the regression fits, with all experimental conditions yielding $R^{2}$ values of at least 0.989.

The effects of nanoparticle addition were quantified relative to DI water at the same temperature. The enhancement ratios for effective CO_2_ uptake and absorption kinetics were defined as

(8) $$E_{q}=\frac{q_{eff,\;NF}-q_{eff,\;DI}}{q_{eff,\;DI}}\times 100\;E_{k}=\frac{k_{app,NF}-k_{app,water}}{k_{app,DI}}\times 100.$$

and the corresponding normalized performance indices were

(9) $$q^{*}=\frac{q_{NF}}{q_{DI}},\;k^{*}=\frac{k_{app,NF}}{k_{app,DI}}$$

where the subscripts NF and DI denote the nanofluid and DI-water conditions, respectively.

For j = q or k, the enhancement ranges across concentrations at fixed temperature and across temperatures at fixed concentration were calculated using Equation (10):

(10) $$R_{c,j}(T)=\underset{c\in C}{\max}E_{j}(T,c)-\underset{c\in C}{\min}E_{j}(T,c),\;R_{T,j}(c)=\underset{T\in T}{\max}E_{j}(T,c)-\underset{T\in T}{\min}E_{j}(T,c).$$

The nanoparticle concentration set, 𝒞, consists of 0.005, 0.01, 0.05, and 0.1 wt%, while the temperature set, 𝒯, consists of 10, 20, and 30 °C. DI water was excluded from 𝒞 because it was used as the reference condition for calculating performance enhancement.

### 2.5. Uncertainty Analysis

Before the CO_2_ absorption experiments, the inlet MFC and outlet MFM were checked under matching flow conditions, and their indicated flow rates agreed. Instrument specifications are summarized in Table 1. Standard uncertainties were estimated from the accuracy limits assuming rectangular distributions:

(11) $$u_{B}(x_{i})=\frac{x_{i,max}-x_{i,min}}{\sqrt{12}}=\frac{a_{i}}{\sqrt{3}}$$

where ai is the half-width of the stated accuracy interval. The specified accuracy of both flow instruments was ±1% of the reading over a flow rate range of 20–80 sccm, corresponding to a relative standard uncertainty of 0.577%.

The standard uncertainty of effective CO_2_ uptake was obtained from the integrated inlet and outlet flows:

(12) $$u_{c}\left(q_{eff}\right)=\frac{M_{CO_{2}}}{V_{m,std}V_{L}}\sqrt{{\left(Au_{r,in}\right)}^{2}+{\left(Bu_{r,out}\right)}^{2}}$$

where A and B are the time-integrated inlet and outlet standard gas volumes, respectively. The relative standard and expanded uncertainties of $q_{eff}$ were 3.36–5.33% and 6.71–10.66%, respectively. The standard and expanded uncertainties of uptake enhancement were 0.1–1.34 and 0.21–2.68 percentage points, respectively.

For $k_{app}$, Monte Carlo gain perturbations were applied to the inlet and outlet flow time series. The perturbed data were then used to recalculate cumulative uptake, normalize the uptake profile, and estimate $k_{app}$ by regression over 0.2 ≤ θ ≤ 0.8. The relative standard and expanded uncertainties of $k_{app}$ were 1.98–4.06% and 3.95–8.12%, respectively. The standard and expanded uncertainties of kinetic enhancement were 0.11–1.92 and 0.21–3.85 percentage points, respectively. These estimates represent the flow-gain component within the instruments’ specified accuracy range.

## 3. Results and Discussion

### 3.1. CO_2_ Uptake Performance

**Figure 6:**
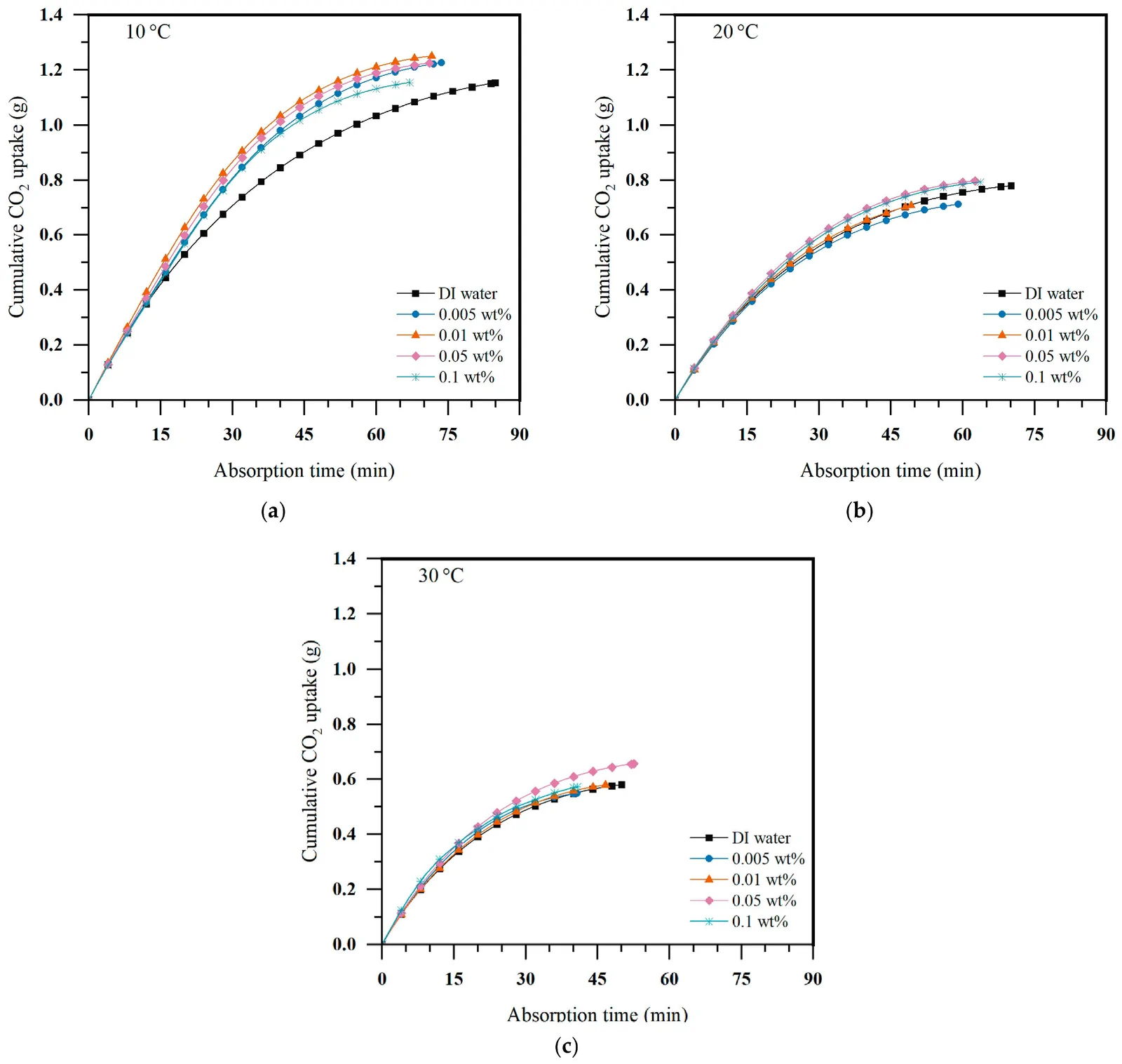
Cumulative CO_2_ uptake as a function of absorption time at different nanoparticle concentrations: (**a**) 10 °C, (**b**) 20 °C, and (**c**) 30 °C.

Figure 6 compares the cumulative CO_2_ uptake at different nanoparticle concentrations at 10 °C, 20 °C, and 30 °C. Under all operating conditions, the cumulative uptake increased rapidly during the initial stage of absorption and then gradually approached a plateau. This behavior reflects the progressive decrease in the inlet–outlet CO_2_ flow-rate difference as the outlet-to-inlet flow-rate ratio approached the termination threshold. The time-dependent profiles also exhibited a clear temperature dependence. At 10 °C, CO_2_ absorption was sustained for a longer period and generally resulted in higher cumulative uptake, whereas the curves at 20 °C and 30 °C approached the termination condition more rapidly. In addition to this temperature effect, the relative positions of the concentration-dependent curves changed with temperature, indicating that the influence of nanoparticle concentration on CO_2_ uptake varied across the investigated thermal conditions.

This concentration-dependent behavior is consistent with trends reported in previous nanofluid-assisted CO_2_ absorption studies. Ansarian and Beiki [14] observed a nonlinear change in the mass-transfer performance of Fe_3_O_4_/water nanofluids in a bubble column, where the enhancement increased over a certain concentration range and subsequently decreased at higher particle loadings. Karamian et al. [22] similarly reported that the CO_2_ absorption characteristics of water-based nanofluids were affected by both nanoparticle concentration and operating conditions. These studies suggest that the concentration effect on CO_2_ absorption should be considered together with the corresponding thermal and hydrodynamic conditions, which is consistent with the variation observed in the cumulative-uptake profiles of Figure 6.

**Figure 7:**
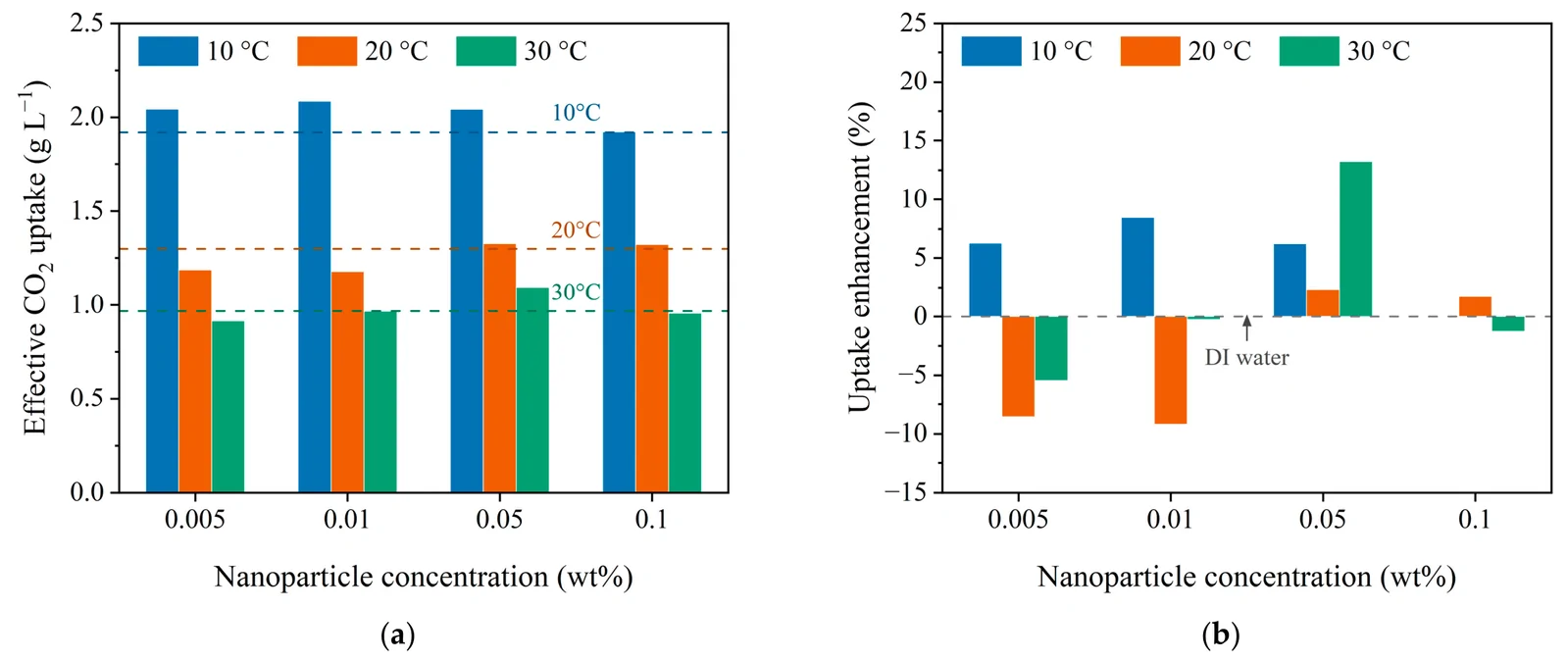
Effects of nanoparticle concentration and temperature on effective CO_2_ uptake: (**a**) effective CO_2_ uptake and (**b**) uptake enhancement relative to DI water.

To quantify these differences more clearly, Figure 7a presents the final effective CO_2_ uptake as a function of nanoparticle concentration and temperature. The most pronounced overall trend was the decrease in effective uptake with increasing temperature. For DI water, the effective uptake decreased from 1.922 g L^−1^ at 10 °C to 1.298 g L^−1^ at 20 °C and 0.967 g L^−1^ at 30 °C, corresponding to an approximately 49.7% reduction between 10 °C and 30 °C. A similar temperature dependence was observed for most nanofluid conditions. This behavior is qualitatively consistent with the temperature-dependent decrease in the equilibrium solubility of CO_2_ in water, as reported and evaluated by Carroll et al. [25].

Within each temperature condition, however, the effect of nanoparticle concentration was nonmonotonic. At 10 °C, the effective uptake increased from 1.922 g L^−1^ for DI water to 2.084 g L^−1^ at 0.01 wt%. In contrast, the highest effective uptake at 20 °C was 1.329 g L^−1^ at 0.05 wt%, while at 30 °C the 0.05 wt% condition also exhibited the highest value of 1.095 g L^−1^, compared with 0.967 g L^−1^ for DI water. Accordingly, the concentration associated with the largest observed effective uptake shifted from 0.01 wt% at 10 °C to 0.05 wt% at both 20 °C and 30 °C. This shift further indicates that the influence of nanoparticle concentration on CO_2_ uptake depends on the operating temperature rather than following a single concentration-dependent trend. Similar variations in the concentration range associated with improved CO_2_ absorption have also been reported under different nanofluid operating conditions [14,22].

The relative effect of nanoparticle addition is shown more directly in Figure 7b, where the effective CO_2_ uptake is compared with the corresponding DI-water baseline at each temperature. At 10 °C, the uptake increased by approximately 6.3%, 8.5%, and 6.2% at 0.005 wt%, 0.01 wt%, and 0.05 wt%, respectively, while the effective uptake at 0.1 wt% remained close to that for DI water. At 20 °C, the 0.05 wt% and 0.1 wt% conditions showed modest enhancements of approximately 2.4% and 1.8%, respectively, whereas the effective uptake at 0.005 wt% and 0.01 wt% decreased by approximately 8.6% and 9.2%, respectively. At 30 °C, the largest uptake enhancement was approximately 13.2% at 0.05 wt%. In contrast, effective CO_2_ uptake decreased by 5.4%, 0.2%, and 1.2% at nanoparticle concentrations of 0.005, 0.01, and 0.1 wt%, respectively, relative to DI water. These results indicate a nonmonotonic concentration dependence whose magnitude and direction vary with temperature under the investigated conditions.

The observed nonmonotonic behavior can be interpreted in terms of temperature-dependent CO_2_ solubility and nanoparticle-mediated mass transfer. The observed decrease in DI-water uptake is consistent with the estimated 42.8% reduction in CO_2_ solubility over the same temperature range [26]. At the maximum nanoparticle concentration of 0.1 wt%, the Einstein model predicts a viscosity increase of only 0.048%, indicating a limited effect of nanoparticle loading on bulk viscosity.

Previous studies have suggested that nanoparticles can enhance CO_2_ absorption through micro-convection, interfacial renewal, and shuttle effects [1,3,6]. Increasing nanoparticle concentration increases the particle number density and nominal surface area available for mass transfer. Based on the TEM-derived mean diameter of 32.7 nm, the theoretical particle number density increases approximately tenfold between 0.01 and 0.1 wt%. However, increased particle interactions can promote cluster formation, potentially reducing particle mobility and accessible surface area. The balance between increased particle availability and aggregation-related limitations may therefore contribute to the observed concentration dependence. These results indicate that CO_2_ uptake is influenced by both temperature-dependent solubility and nanoparticle concentration, with the latter potentially affecting particle-mediated transport behavior.

### 3.2. Effect of Temperature and Nanoparticle Concentration on CO_2_ Absorption Kinetics

**Figure 8:**
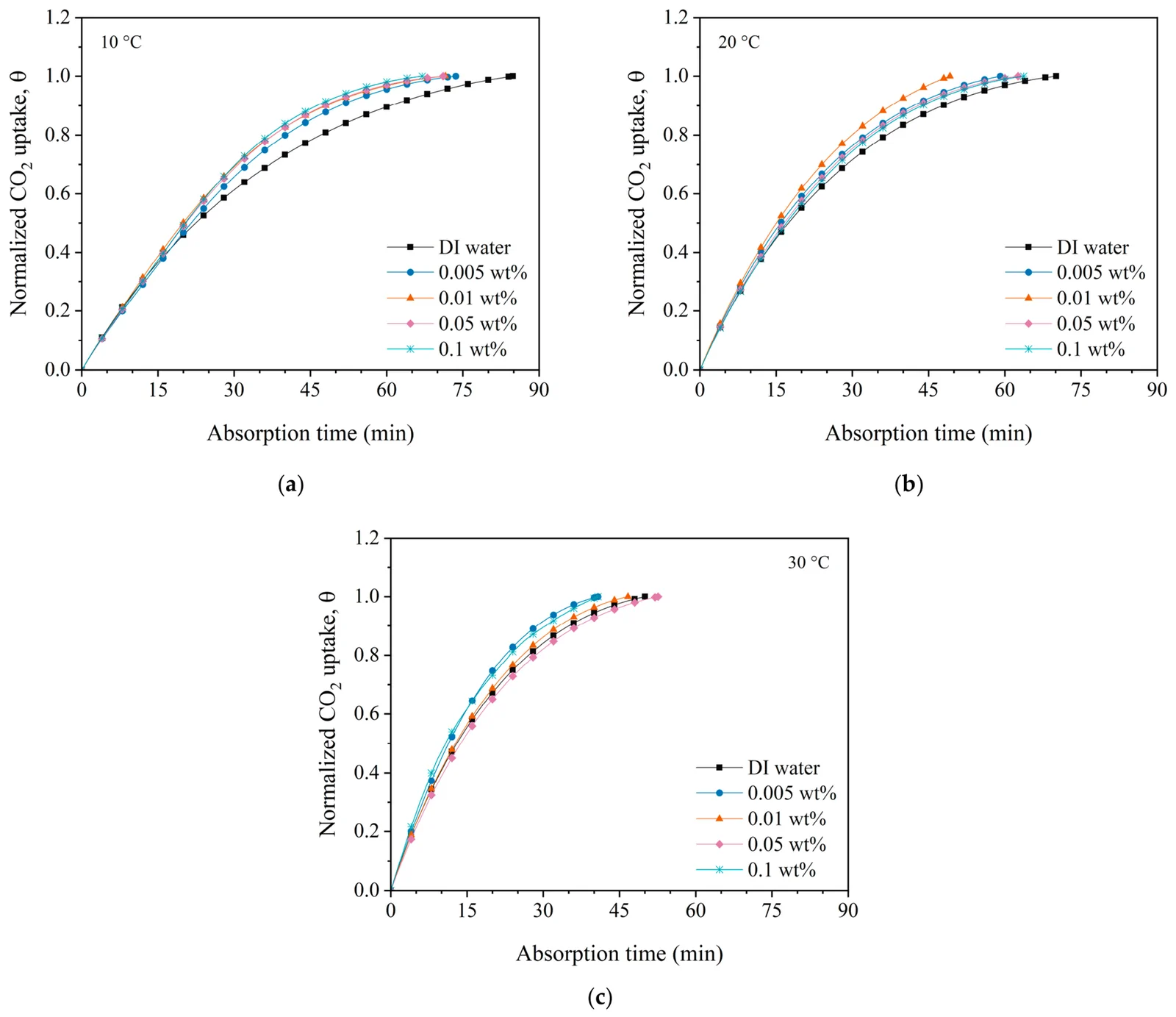
Normalized CO_2_ uptake, θ, as a function of time at different nanoparticle concentrations: (**a**) 10 °C, (**b**) 20 °C, and (**c**) 30 °C.

Figure 8 compares the normalized CO_2_ uptake profiles at different nanoparticle concentrations and temperatures. Because the cumulative uptake was normalized by the final effective uptake of each condition, these profiles emphasize the temporal progression of absorption independently of differences in the final uptake level. For DI water, the times required to reach 50% of the final effective uptake were approximately 1345 s, 1045 s, and 778 s at 10 °C, 20 °C, and 30 °C, respectively, while the corresponding times to reach 80% were approximately 2818 s, 2203 s, and 1624 s. Thus, although the final effective CO_2_ uptake decreased with increasing temperature, the relative absorption progress became faster at higher temperatures.

Nanoparticle addition also modified the normalized uptake behavior, and the magnitude of this effect varied with concentration and temperature. At 10 °C and 20 °C, the nanofluid profiles generally reached a given normalized uptake level earlier than the corresponding DI water profiles, consistent with faster absorption kinetics. At 30 °C, the response became more concentration dependent: the 0.005 wt% and 0.1 wt% conditions showed faster normalized uptake, whereas normalized uptake at 0.05 wt% progressed at a rate comparable to or slightly lower than that for DI water. These trends indicate that the rate at which the final effective uptake is approached does not necessarily follow the same concentration dependence as the final uptake itself.

Previous studies have also reported enhanced gas–liquid transport in Fe_3_O_4_-based nanofluids. Pahlevaninezhad et al. [16] observed an increase in the liquid-side mass-transfer coefficient of Fe_3_O_4_/water nanofluids in a magnetic-field-assisted falling-film system, with maximum enhancements of 111% and 13.7% under turbulent and laminar conditions, respectively. Karimi Darvanjooghi et al. [17] similarly reported that the application of a magnetic field to Fe_3_O_4_/water nanofluids increased CO_2_ mass diffusivity and the surface-renewal factor while reducing the diffusion-layer thickness, resulting in increased molar flux. Although these studies were conducted under magnetic-field-assisted conditions, their results demonstrate that Fe_3_O_4_ nanoparticles can modify liquid-side and interfacial transport processes, providing a relevant basis for interpreting the changes in dynamic absorption behavior observed in Figure 8.

Nanoparticle concentration also affected $k_{app}$, and its influence varied with temperature. At 10 °C, $k_{app}$ increased for all nanofluid concentrations relative to DI water, with the highest value observed at 0.1 wt%. At 20 °C, all tested nanofluids also exhibited higher $k_{app}$ values, with the maximum occurring at 0.01 wt%. At 30 °C, the concentration dependence was mixed: the largest $k_{app}$ was observed at 0.005 wt%, whereas the value at 0.05 wt% was slightly lower than that of DI water. These results show that the kinetic response to nanoparticle addition is nonmonotonic and changes with operating temperature.

**Figure 9:**
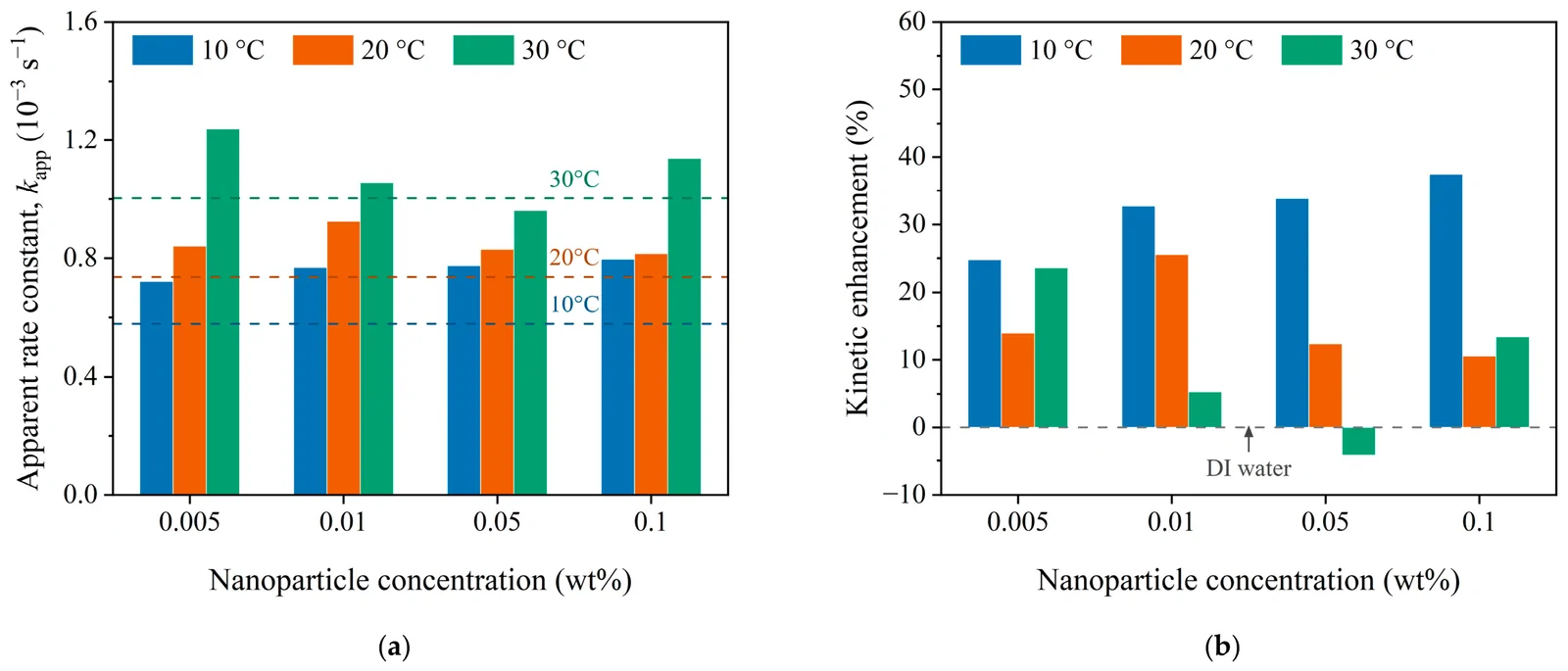
Effects of nanoparticle concentration and temperature on CO_2_ absorption kinetics: (**a**) apparent absorption rate constant and (**b**) kinetic enhancement relative to DI water.

This behavior is shown more clearly in Figure 9b, which compares the enhancement in $k_{app}$ relative to DI water at the same temperature. At 10 °C, the kinetic enhancement ranged from approximately 24.8% to 37.5%, with the largest increase at 0.1 wt%. At 20 °C, enhancements of approximately 10.6–25.5% were obtained, with the largest increase at 0.01 wt%. At 30 °C, $k_{app}$ increased by approximately 23.5%, 5.3%, and 13.5% at 0.005 wt%, 0.01 wt%, and 0.1 wt%, respectively, whereas the 0.05 wt% condition showed an approximately 4.1% decrease. Thus, positive kinetic enhancement was observed under most of the investigated concentration–temperature conditions, but the concentration giving the largest enhancement differed with temperature. Overall, the kinetic results showed that increasing temperature led to a more rapid approach to the final uptake, as evidenced by shorter normalized uptake times and higher $k_{app}$ values for DI water. However, the additional kinetic effect of APTES-modified Fe_3_O_4_ was strongly dependent on both nanoparticle concentration and temperature. The largest kinetic enhancement was approximately 37.5% at 0.1 wt% and 10 °C, compared with approximately 25.5% at 0.01 wt% and 20 °C and 23.5% at 0.005 wt% and 30 °C. In contrast, the 0.05 wt% condition, which enhanced effective CO_2_ uptake at all three temperatures, exhibited progressively smaller kinetic enhancement with increasing temperature and showed a slight decrease in $k_{app}$ at 30 °C. These results indicate that effective CO_2_ uptake and apparent absorption kinetics exhibited different responses to nanoparticle addition under the investigated conditions.

### 3.3. Relationship Between CO_2_ Uptake and Absorption Kinetics

**Figure 10:**
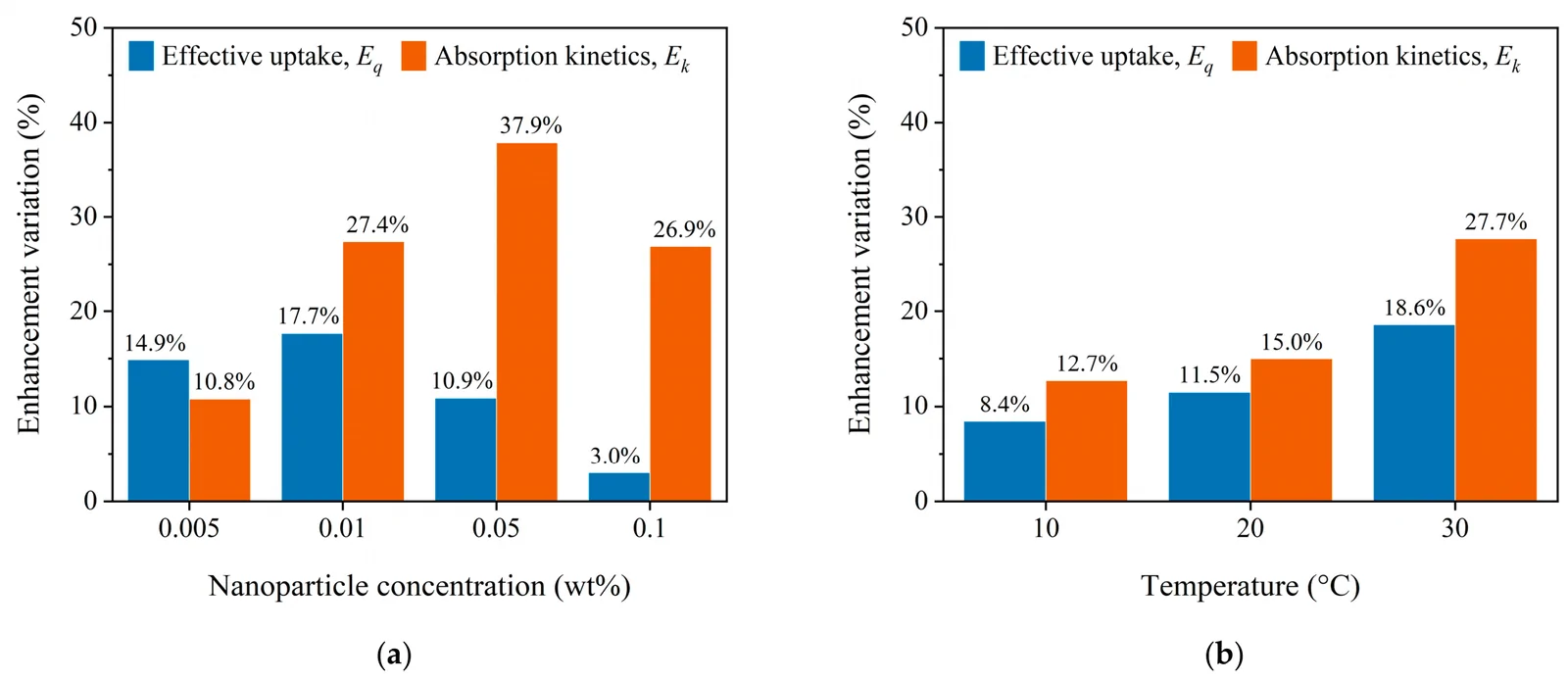
Enhancement variation of effective CO_2_ uptake and absorption kinetics: (**a**) concentration dependence; (**b**) temperature dependence.

To further characterize the dependence of the observed performance changes on nanoparticle concentration and temperature, Figure 10 compares the ranges of effective CO_2_ uptake enhancement, ($E_{q}$), and kinetic enhancement, ($E_{k}$), both referenced to DI water at the corresponding temperature. For each metric, the enhancement range was defined as the difference between the maximum and minimum enhancement values obtained by varying one parameter while holding the other constant. Figure 10a therefore describes the variation across nanoparticle concentrations at each temperature, whereas Figure 10b describes the variation across temperatures at each concentration.

As shown in Figure 10a, the concentration-dependent range of $E_{q}$ increased from 8.4% at 10 °C to 11.5% at 20 °C and 18.6% at 30 °C. The corresponding ranges of $E_{k}$ were 12.7%, 15.0%, and 27.7%, respectively. Thus, the spread of enhancement values across the tested concentrations became wider at higher temperatures for both metrics. Moreover, $E_{k}$ exhibited a larger concentration-dependent range than $E_{q}$ at every temperature, indicating a stronger response of the relative kinetic performance to concentration changes. These results show that the extent of concentration dependence itself varied with operating temperature, with the greatest differentiation among concentrations observed at 30 °C.

Figure 10b shows that the temperature-dependent ranges varied nonmonotonically with nanoparticle concentration. For $E_{q}$, the range increased from 14.9% at 0.005 wt% to 17.7% at 0.01 wt%, before decreasing to 10.9% at 0.05 wt% and 3.0% at 0.1 wt%. For $E_{k}$, the corresponding ranges were 10.8%, 27.4%, 37.9%, and 26.9%. The largest temperature-dependent variation therefore occurred at 0.01 wt% for uptake enhancement and at 0.05 wt% for kinetic enhancement. The range of $E_{k}$ exceeded that of $E_{q}$ at concentrations of 0.01–0.1 wt%, while the opposite relationship was observed at 0.005 wt%. In particular, at 0.1 wt%, the relatively narrow range of $E_{q}$ was accompanied by a much wider range of $E_{k}$. This result demonstrates that limited variation in uptake enhancement with temperature does not necessarily imply a similarly limited variation in kinetic enhancement.

Taken together, these results indicate that the relative uptake and kinetic responses to APTES-modified Fe_3_O_4_ addition exhibit distinct dependencies on concentration and temperature. The widening concentration-dependent ranges at higher temperatures, together with the different concentration dependence of the temperature-related ranges, support evaluating nanoparticle concentration and operating temperature jointly.

**Figure 11:**
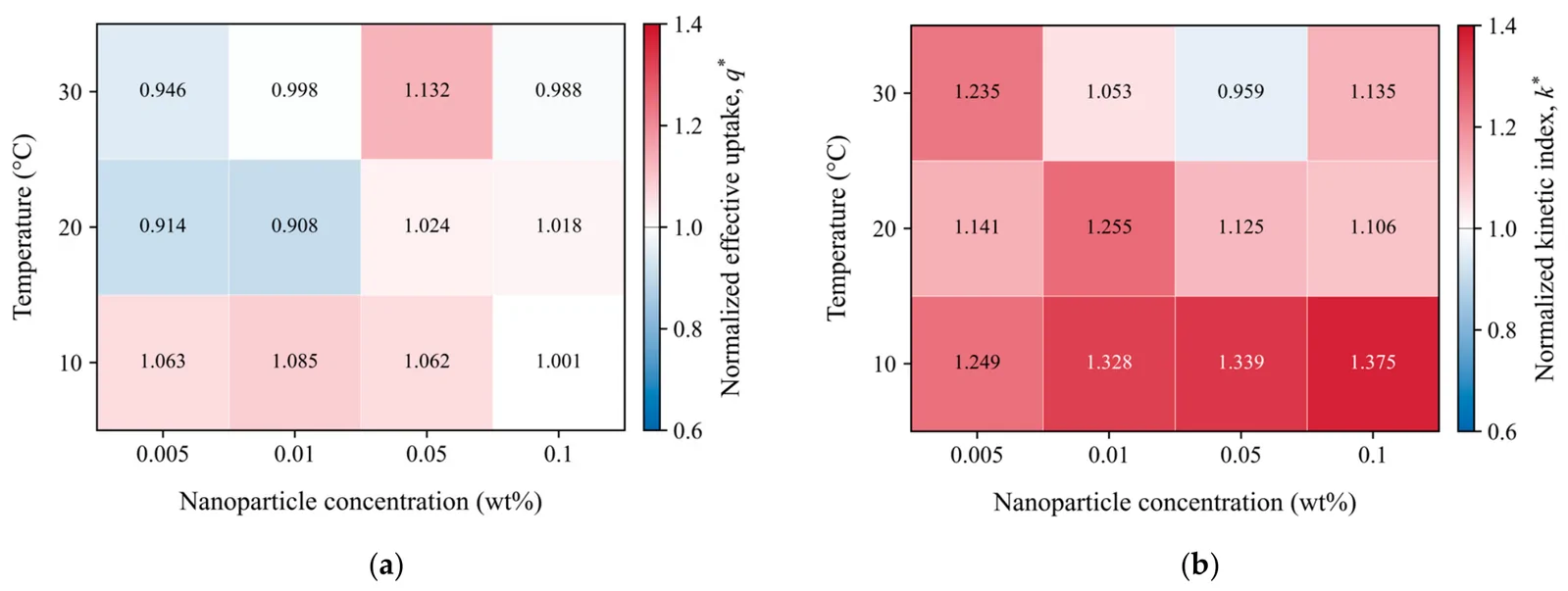
Concentration–temperature performance maps of normalized CO_2_ absorption characteristics: (**a**) normalized effective uptake and (**b**) normalized kinetic index.

The combined behavior of these two performance metrics is shown more directly in Figure 11 through concentration–temperature maps of the normalized effective uptake and normalized kinetic index. At 10 °C, both indices were at or above unity for all nanofluid concentrations. At 0.01 wt%, for example, the normalized effective uptake and kinetic index were 1.085 and 1.328, respectively. At 20 °C, the 0.005 wt% and 0.01 wt% conditions exhibited normalized effective uptake below unity while maintaining kinetic indices above unity, whereas the 0.05 wt% and 0.1 wt% conditions showed modest improvement in both metrics.

At 30 °C, the contrast between uptake and kinetics became more apparent. The 0.005 wt% condition showed a normalized effective uptake of 0.946 and a kinetic index of 1.235, whereas the 0.05 wt% condition showed the opposite response, with values of approximately 1.132 and 0.959, respectively. The 0.1 wt% condition maintained an effective uptake close to the DI-water baseline while increasing the kinetic index to 1.135. These results show that nanoparticle concentration does not influence effective uptake and absorption kinetics in the same manner, and the balance between the two metrics changes with temperature. Similar concentration-dependent changes in CO_2_ absorption and mass-transfer behavior have been associated with nanoparticle concentration, dispersion characteristics, and particle size or hydrodynamic diameter in previous studies [14,15,22].

The performance maps suggest that nanoparticle concentration and operating temperature should be considered together when evaluating CO_2_ uptake and absorption kinetics. Under the investigated conditions, increasing temperature was associated with lower effective CO_2_ uptake and faster normalized absorption, while the effect of nanoparticle addition varied with concentration. Conditions exhibiting higher effective uptake may be favorable when the absorbed CO_2_ amount per unit liquid volume is prioritized, whereas higher apparent absorption rate constants may be relevant when faster absorption is desired. However, CO_2_ desorption, absorbent regeneration, nanoparticle recovery, and stability over repeated absorption–regeneration cycles were not evaluated in this study. These findings therefore provide preliminary information rather than definitive practical operating recommendations.

Overall, the integrated analysis suggests that APTES-modified Fe_3_O_4_ addition is associated with different uptake and kinetic responses across the investigated nanoparticle concentrations and operating temperatures. Effective uptake showed a pronounced temperature dependence, whereas the effect of nanoparticle addition on absorption kinetics varied among conditions. The normalized performance maps further suggest that conditions associated with higher uptake do not necessarily yield the greatest kinetic enhancement.

## 4. Conclusions

This study experimentally investigated the effects of APTES-modified Fe_3_O_4_ nanoparticle concentration (0–0.1 wt%) and temperature (10–30 °C) on CO_2_ uptake and absorption kinetics under continuous bubbling conditions. For DI water, effective CO_2_ uptake decreased from 1.922 g L^−1^ at 10 °C to 0.967 g L^−1^ at 30 °C, corresponding to a 49.7% reduction, whereas normalized absorption progressed more rapidly at higher temperatures. The highest effective uptake of 2.084 g L^−1^ was obtained at 10 °C and 0.01 wt%, while the maximum uptake enhancement relative to DI water was 13.2% at 30 °C and 0.05 wt%. The greatest kinetic enhancement was 37.5% at 10 °C and 0.1 wt%. Notably, at 30 °C and 0.05 wt%, effective uptake increased by 13.2%, whereas the apparent absorption rate constant decreased by 4.1%, indicating that these two performance metrics did not improve simultaneously under this condition. The concentration-dependent enhancement ranges increased from 8.4% to 18.6% for effective uptake and from 12.7% to 27.7% for absorption kinetics as temperature increased from 10 °C to 30 °C. Across temperatures, the largest uptake enhancement range was 17.7% at 0.01 wt%, whereas the largest kinetic enhancement range was 37.9% at 0.05 wt%. These findings suggest that CO_2_ uptake and absorption kinetics exhibit different concentration–temperature dependencies over the investigated ranges. Both metrics may inform preliminary comparisons of operating conditions, although practical recommendations require further evaluation of regeneration, desorption, nanoparticle recovery, and cycling stability.

## Figures and Tables

**Table 1:** Specifications of the measuring instruments used in the CO_2_ absorption experiments.

Instrument	Device	Measurement Range	Accuracy
Mass flow controller	WIZ-701C-LT-1(KMB Tech.)	0–80 sccm	±1.0% RD (25–100% FS)
Mass flow meter	WIZ-701M-LT-1(KMB Tech.)	0–80 sccm	±1.0% RD (25–100% FS)
Temperature sensor	RTD Pt 100	−200–600 °C	±0.15 °C
Analytical balance	A&D/GH-200	0–220 g	±0.001 g

## Data Availability

The original contributions presented in this study are included in the article. Further inquiries can be directed to the corresponding author.
